# Infantile extreme hypertriglyceridemia diagnosed as glycogen storage disease type Ia: A case report

**DOI:** 10.1097/MD.0000000000047959

**Published:** 2026-03-20

**Authors:** Chuanjie Yuan, Ying Liu, Juanjuan Lyu, Xiaomei Sun, Jin Wu

**Affiliations:** a Department of Pediatrics, West China Second University Hospital, Sichuan University, Chengdu, China; b Key Laboratory of Birth Defects and Related Diseases of Women and Children (Sichuan University), Ministry of Education, Chengdu, China.

**Keywords:** glycogen storage disease, hypertriglyceridemia, type Ia

## Abstract

**Rationale:**

Glycogen storage disease type Ia (GSD Ia) typically presents with fasting hypoglycemia and hyperlipidemia. Atypical infantile presentations can delay diagnosis. We report an infant with extreme hypertriglyceridemia ultimately diagnosed as GSD Ia.

**Patient concerns:**

A 5-month-old girl presented with poor appetite, growth retardation, hepatomegaly, and extreme hypertriglyceridemia (72 mmol/L), hypercholesterolemia, elevated transaminases, hyperuricemia, and hyperlactatemia, but initial normal blood glucose.

**Diagnoses:**

Initial differentials included familial hypertriglyceridemia, but metabolic screening was normal. Whole-exome sequencing confirmed GSD Ia with compound heterozygous *G6PC* mutations (c.648G>T and c.814G>T).

**Interventions:**

Initial lipid-lowering (low-fat diet, fenofibrate, omega-3, plasma exchange) reduced triglyceride. Post-diagnosis, she received lactose-free formula with frequent feeds and nocturnal nutrition; uncooked cornstarch was introduced at 6 months, though adherence was initially poor.

**Outcomes:**

Metabolic control was unstable until age 2 due to poor adherence. After 24 months of structured cornstarch therapy, fasting glucose normalized (4.2–6.6 mmol/L), triglycerides decreased (1.8–6.7 mmol/L), and catch-up growth occurred (height Z-score from -3.9 to -2.2 by 36 months).

**Lessons:**

GSD Ia should be considered in infantile extreme hypertriglyceridemia with growth retardation, even without classic hypoglycemia. Sustained metabolic control requires multidisciplinary strategies addressing both biochemical and adherence barriers.

## 1. Introduction

GSD Ia is a glucose metabolic disorder caused by a deficiency of the G6Pase-α that is essential for maintaining glucose homeostasis.^[1]^ It typically manifests as a range of metabolic complications, including fasting hypoglycemia, lactic acidosis, hypertriglyceridemia, and hyperuricemia. As is well documented, these metabolic disturbances can lead to long-term complications such as hepatic steatosis, hepatocellular adenomas, and renal dysfunction.^[1]^ In young infants, the initial symptoms are generally atypical, such as feeding difficulties or apnea. In contrast, older infants may also present with a failure to thrive and a protruding abdomen. Other common clinical presentations include doll-like facial appearance, growth failure, developmental delay, and hypotrophic musculature.^[2]^ Here, we report the case of a female infant who presented with extreme hypertriglyceridemia and was ultimately diagnosed with GSD Ia during treatment.

## 2. Case description

The patient was born to a non-consanguineous couple with a birth weight of 3000 g and a birth length of 50 cm. There was no family history of liver or metabolic disease. She was artificially fed–600 to 800 mL milk daily after birth. At 3 months of age, the patient presented with a poor appetite and slow growth in height and weight. In contrast, no episodes of vomiting, choking, abdominal distension, or diarrhea were reported. A child health specialist advised the patient to seek medical attention at 5 months of age. Upon admission, physical examination revealed a weight of 5.1 kg (length-for-age percentile 0.7) and length of 58.1 cm (length-for-age percentile 0.4). Thin subcutaneous fat and a poor nutritional status were observed. Superficial lymph nodes were not enlarged. In addition, the abdomen was soft and the liver was palpable at 2.5 cm below the right costal margin and 3.0 cm below the sinew margin. The spleen was not palpable below the left costal margin, and the heart, lungs, and neurological examination findings were unremarkable.

**Figure 1.**
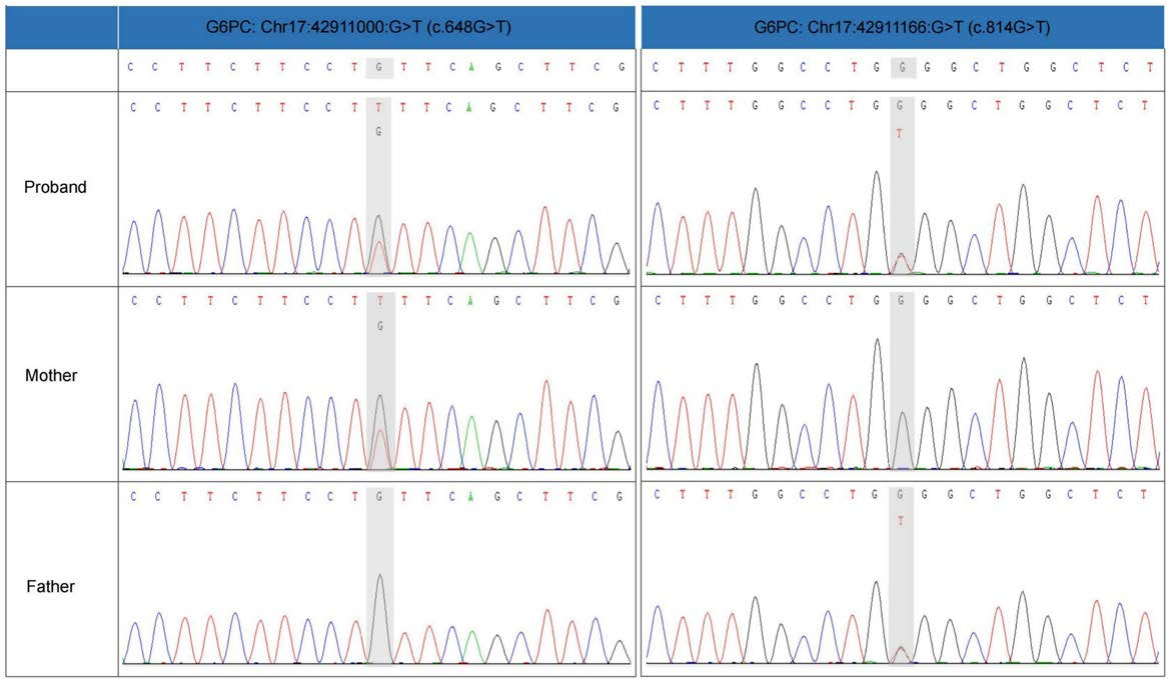
Sanger sequencing results validated the G6PC gene mutation in the proband and her parents. Sequencing traces are displayed for the reference sequence, proband, mother, and father, highlighting heterozygous mutations in the proband and their inheritance patterns. G6PC = glucose-6-phosphatase catalytic subunits.

The patient exhibited significantly elevated blood lactate levels (5.7 mmol/L) while maintaining normal pH and glucose levels. There was also evidence of severe transaminitis, with alanine aminotransferase (ALT) at 285 U/L and aspartate aminotransferase (AST) at 349 U/L, alongside profound hyperlipidemia, indicated by triglycerides (TG) at 72.0 mmol/L and total cholesterol at 15.07 mmol/L. Additional laboratory findings included hyperuricemia (464 µmol/L), elevated pyruvate (319.60 µmol/L), β-hydroxybutyrate (0.82 mmol/L), and free fatty acids (0.88 mmol/L) (Table 1). An abdominal ultrasound revealed mild hepatomegaly. The initial differential diagnoses considered familial hypertriglyceridemia or chylomicronemia, while inherited metabolic disorders were also contemplated. Analysis of plasma amino acids, acylcarnitines, and urinary organic acids revealed normal results. Whole-exome sequencing identified compound heterozygous mutations in the G6PC gene (c.648G >T and c.814G >T) (Fig. 1), confirming a diagnosis of GSD Ia. The c.648G >T splice-site variant is prevalent in Chinese populations, whereas the c.814G >T (p.G272W) variant is rare and predicted to be pathogenic,^[3]^ with an Alpha Missense score of 0.942.^[4]^

**Table 1:** Laboratory findings of the patient with GSD Ia.

Text index (unit)	1st day of hospitalization	14th day of hospitalization	Reference
White blood cell count (10^9^/L)	17.1	–	4.3–14.2
Absolute neutrophil count (10^9^/L)	3.29	–	0.6–7.5
PH	7.46	–	7.35–7.45
Lactic acid (mmol/L)	5.7	–	0.7–3.0
Blood glucose (mmol/L)	5.3	1.1	3.5–5.5
Alanine aminotransferase (U/L)	285	73	0–49
Aspartate aminotransferase (U/L)	349	83	0–40
Uric acid (µmol/L)	464	372	180–360
Total cholesterol (mmol/L)	15.07	6.67	<5.18
Triglycerides (mmol/L)	72	14.3	<1.76
High-density lipoprotein cholesterol (mmol/L)	2.06	0.46	>1.0
Low-density lipoprotein cholesterol (mmol/L)	2.2	0.9	<3.3
Apolipoprotein A1 (g/L)	0.24	0.63	0.76–2.14
Apolipoprotein B (g/L)	0.3	0.9	0.46–1.42
Pyruvate (µmol/L)	319.6	–	20–100
β-hydroxybutyrate (mmol/L)	0.82	–	0–0.27
Free fatty acid (mmol/L)	0.88	–	0.1–0.77

GSD Ia = glycogen storage disorder type Ia.

**Figure 2.**
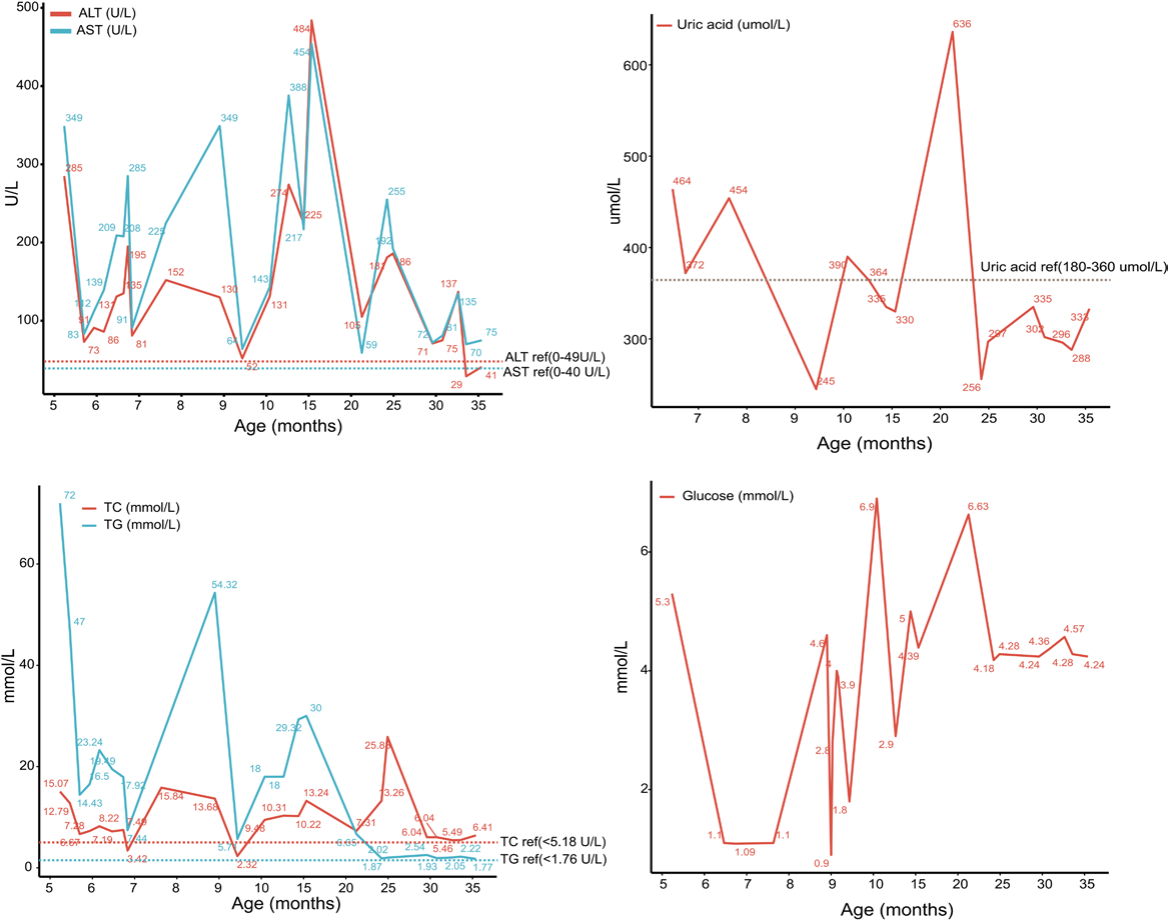
Longitudinal changes in biochemical parameter levels of the patient from 5 to 36 months of age. (A) ALT and AST levels; (B) uric acid levels (µmol/L); (C) TC and TG levels (mmol/L); (D) glucose levels (mmol/L). ALT = alanine aminotransferase, AST = aspartate aminotransferase, TC = total cholesterol, TG = triglyceride.

**Figure 3.**
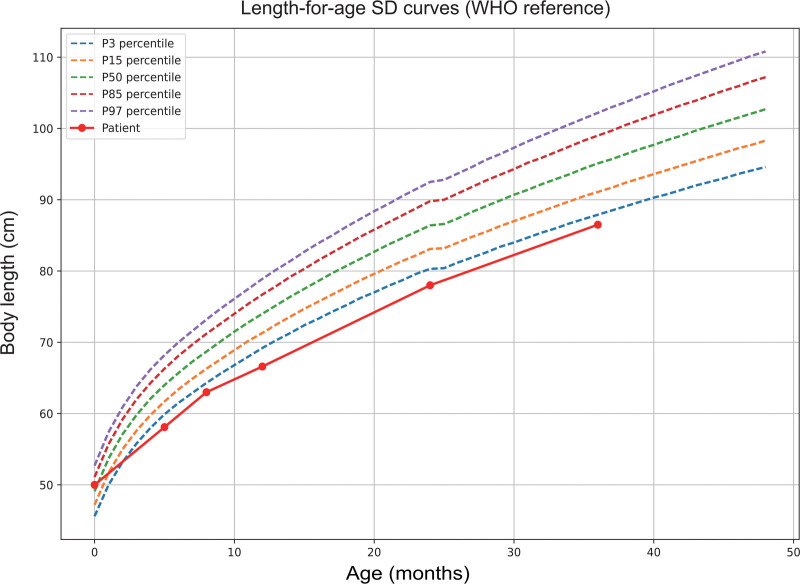
Body length distribution of the patient during follow-up compared to WHO reference length-for-age SD curves. The patient’s growth trajectory (solid red line) is plotted against the WHO percentile curves: P3, P15, P50, P85, and P97 percentiles (dashed lines). Body length was measured in centimeters over age in months. SD = standard deviation.

Initial treatment involved a low-fat diet, fenofibrate, omega-3 fatty acids, and plasma exchange, which effectively reduced triglyceride levels and transaminases but resulted in hypoglycemia (Table 1). Following diagnosis, the patient was transitioned to a lactose-free formula with frequent feedings and nocturnal enteral nutrition. At 6 months, uncooked cornstarch (UCCS, 1.5 g/kg 4 times daily) was introduced; however, adherence was inconsistent due to poor tolerance and issues with parental compliance. Consequently, recurrent episodes of hypoglycemia, hypertriglyceridemia (5.71–54.32 mmol/L), hyperuricemia (245–636 μmol/L), and transaminitis (ALT 52–484 U/L, AST 64–454 U/L) persisted until the age of 2 (Fig. 2). Following the structured implementation of UCCS over 24 months, there was a notable improvement in metabolic control, evidenced by normalized fasting glucose levels ranging from 4.18 to 6.63 mmol/L, and a reduction in hyperlipidemia, with triglyceride levels decreasing to between 1.77 and 6.65 mmol/L (Fig. 2). Additionally, catch-up growth was observed, as indicated by an increase in the height-for-age Z-score from −3.9 to −2.2 by the 36-month mark (Fig. 3).

## 3. Discussion

Severe hypertriglyceridemia in infants can be a challenging condition for diagnosis and management because of its rarity and potential for severe complications. The etiology of hypertriglyceridemia in children is multifactorial, encompassing both secondary and primary causes. Secondary factors such as obesity, insulin resistance, diabetes mellitus, hypothyroidism, nephrotic syndrome, and medications (e.g, glucocorticoids and retinoids) are frequently implicated.^[5]^ However, in infants, particularly those presenting with extreme hypertriglyceridemia (TG levels >1000 mg/dL), the underlying etiology is more likely to be primary or genetic. Notably, extreme hypertriglyceridemia in infancy is strongly associated with genetic defects that affect lipoprotein lipase (LPL)-mediated TG clearance. According to an earlier study, biallelic pathogenic variants in LPL, Apolipoprotein C-Ⅱ(APOC2), Apolipoprotein A-V (APOA5), Glycosylphosphatidylinositol-anchored high-density lipoprotein-binding protein 1 (GPIHBP1), and lipase maturation factor 1 disrupt the lipolytic cascade.^[6]^ The patient exhibited no specific clinical manifestations, with poor appetite and growth restriction as the primary clinical symptoms at the time of referral. Laboratory investigations revealed severe hypertriglyceridemia and hypercholesterolemia, which suggested a possible inherited lipid metabolism disorder. Nevertheless, congenital lipid metabolism disorders do not typically present with hepatomegaly, hyperuricemia, or hyperlactataemia. Initially, the patient did not exhibit significant hypoglycemia, which is a key diagnostic indicator. Therefore, diagnosing the condition in its early stages is challenging. However, the later discovery of hypoglycemia, along with previously observed symptoms, was highly indicative of glycogen storage disease. Combined with the observed metabolic abnormalities, these findings allowed for a more targeted investigation and ultimately pointed toward a diagnosis of glycogen storage disorder.

GSD Ia is caused by a deficiency in the G6Pase complex, resulting in excessive accumulation of glycogen and fat in the liver, kidney, and intestinal mucosa. Patients with GSD Ia display a wide spectrum of clinical manifestations, including hepatomegaly, hypoglycemia, lactic acidemia, hyperlipidemia, hyperuricemia, and growth retardation.^[7]^ The mechanism by which GSD Ia induces hyperlipidemia remains elusive but may result from accelerated glycolysis, generating excess acetyl-CoA for lipid production. This leads to an increased lipid release into the bloodstream and reduced lipid clearance. Previous studies have documented a 40-fold increase in palmitate synthesis and a 7-fold increase in cholesterol levels in patients with GSD Ia.^[8]^ However, reports of extreme hypertriglyceridemia in infants with GSD Ia are limited. Sever et al reported a case of infantile-onset GSD Ia with hypotonia, undetectable serum glucose, hyperlactatemia, and extreme hypertriglyceridemia (TG >5000 mg/dL).^[9]^ Moreover, Fang et al reported a case of infantile-onset GSD Ia with hypoglycemia, hyperlactatemia, and extreme hyperlipidemia (TG levels: 2772.3 mg/dL).^[10]^ It is noteworthy that the present case exhibited similarities with the 2 previously mentioned cases in that typical symptoms were absent at the onset and the presence of extreme hypertriglyceridemia. However, the present case did not initially exhibit hallmark hypoglycemia, which posed a diagnostic challenge.

Patients with severe hypertriglyceridemia may experience recurrent episodes of acute pancreatitis, hepatosplenomegaly, lipemia retinalis, or eruptive xanthoma. Meanwhile, high serum triglyceride levels in childhood in GSD Ia are associated with an increased risk of hepatocellular adenoma later in life.^[11,12]^ In this case, the patient required rapid reduction in triglyceride levels to prevent complications such as pancreatitis. Thus, the patient was managed with dietary adjustments, fenofibrate, oral omega-3 polyunsaturated fatty acids, and plasma exchange therapy, which yielded favorable outcomes. However, the TG levels subsequently increased on multiple occasions. The most effective treatment for lipid management in patients with GSD Ia is to maintain stable blood glucose levels. Moreover, maintaining blood glucose levels ≥3.9 mmol/l (70 mg/dL) is crucial for achieving satisfactory metabolic control.^[7]^ The most efficacious modality at present is dietary management, including multiple lactose-free formula feedings during infancy, as well as monitoring blood glucose levels and feeding every 3 to 4 hours during the night. After 6 months of age, the addition of UCCS has been established to provide a steady release of glucose, thereby stabilizing glucose levels over an extended period.^[2]^ Dietary treatment is the cornerstone of the GSD Ia treatment. This case highlights the significant challenges linked to dietary treatment during infancy, characterized by nonadherence to nocturnal feeding schedules and gastrointestinal intolerance to UCCS. Initial recommendations for nocturnal nasogastric tube feeding to ensure glycemic stability were declined by the guardians because of concerns regarding procedural distress, resulting in recurrent hypoglycemic episodes. These observations emphasize the critical need for enhanced coordination of long-term ambulatory care and systematic parental education programs. More importantly, implementing a multidisciplinary transitional care model incorporating structured parental education curricula on metabolic emergency prevention and psychosocial support interventions may optimize adherence to complex dietary regimens in pediatric GSD Ia management.

In summary, this case highlights an infant with extreme hypertriglyceridemia who was diagnosed with GSD Ia. Severe hypertriglyceridemia warrants an expanded genetic/metabolic evaluation, even in the absence of classic hypoglycemia. Therefore, GSD Ia should be considered in the differential diagnosis of infantile primary hypertriglyceridemia with growth retardation. Finally, achieving sustained metabolic control requires multidisciplinary strategies addressing both biochemical derangements and barriers to treatment adherence.

## Acknowledgments

The authors express profound appreciation to all study investigators and patients for their invaluable contributions to this research.

## Author contributions

**Conceptualization:** Chuanjie Yuan, Ying Liu, Juanjuan Lyu, Jin Wu.

**Investigation:** Chuanjie Yuan, Ying Liu, Juanjuan Lyu.

**Project administration:** Chuanjie Yuan, Juanjuan Lyu, Xiaomei Sun.

**Resources:** Chuanjie Yuan, Xiaomei Sun, Jin Wu.

**Supervision:** Xiaomei Sun.

**Writing – original draft:** Chuanjie Yuan, Ying Liu, Jin Wu.

**Writing – review & editing:** Chuanjie Yuan, Ying Liu, Jin Wu.
